# Chromosomal Diversification in *Ancistrus* Species (Siluriformes: Loricariidae) Inferred From Repetitive Sequence Analysis

**DOI:** 10.3389/fgene.2022.838462

**Published:** 2022-03-24

**Authors:** Kevin Santos da Silva, Larissa Glugoski, Marcelo Ricardo Vicari, Augusto César Paes de Souza, Renata Coelho Rodrigues Noronha, Julio Cesar Pieczarka, Cleusa Yoshiko Nagamachi

**Affiliations:** ^1^ Laboratório de Citogenética, Centro de Estudos Avançados da Biodiversidade, Instituto de Ciências Biológicas, Universidade Federal do Pará, Belém, Brazil; ^2^ Laboratório de Citogenética de Peixes, Universidade Federal de São Carlos, São Carlos, Brazil; ^3^ Laboratório de Biologia Cromossômica: Estrutura e Função, Departamento de Biologia Estrutural, Molecular e Genética, Universidade Estadual de Ponta Grossa, Ponta Grossa, Brazil; ^4^ Laboratório de Estudo da Ictiofauna Amazônica, Instituto Federal de Educação, Ciência e Tecnologia do Pará, Abaetetuba, Brazil

**Keywords:** amazon fish, comparative cytogenetics, microsatellites, repetitive DNA, rDNA, sex chromosomes

## Abstract

The *Ancistrus* genus has extensive chromosomal diversity among species, including heteromorphic sex chromosomes occurrence. However, studies have been shown that chromosomal diversity may still be underestimated. Repetitive sequences represent a large part of eukaryotic genomes, associated with mechanisms of karyotypic diversification, including sex chromosomes evolution. This study analyzed the karyotype diversification of two *Ancistrus* species (*Ancistrus* sp. 1 and *Ancistrus* sp. 2) from the Amazon region by classical and molecular chromosomal markers. Conventional chromosome bands and fluorescence *in situ* hybridization using probes 18S and 5S rDNA, besides (CA)n, (CG)n, (GA)n, (CAC)n, (CAG)n, (CAT)n, (GAA)n, (GAC)n, (TAA)n, and (TTAGGG)n in tandem repeats were determined on the karyotypes. *Ancistrus* sp. 1 and *Ancistrus* sp. 2 presented karyotypes with 2n = 38 (20 m + 14sm+4st, XX/XY) and 2n = 34 (20 m + 14sm, without heteromorphic sex chromosomes), respectively. Robertsonian rearrangements can explain the diploid number difference. C-bands occurred in pericentromeric regions in some chromosomes, and a single 18S rDNA locus occurred in both species. The 5S rDNA showed variation in the number of loci between species karyotypes, suggesting the occurrence of unstable sites and rearrangements associated with these sequences in *Ancistrus.* The microsatellite mapping evidenced distinct patterns of organization between the two analyzed species, occurring mainly in the sex chromosomes in *Ancistrus* sp. 1, and in the centromeric and pericentromeric regions of chromosomes m/sm in *Ancistrus* sp. 2. These data shows the extensive chromosomal diversity of repetitive sequences in *Ancistrus*, which were involved in Robertsonian rearrangements and sex chromosomes differentiation.

## Introduction

Loricariidae is one of the most specious neotropical fish families of the Siluriformes order, having 1,023 species (Fricke et al., 2021) distributed throughout Central and South America, from southeastern Costa Rica to northeastern Argentina (Armbruster, 2004; Armbruster, 2008; Armbruster and Lujan, 2016). Loricariidae is a monophyletic group organized into six subfamilies: Lithogeninae, Delturinae, Hypoptopomatinae, Neoplecostominae, Loricariinae, and Hypostominae (Roxo et al., 2019). The subfamily Hypostominae is the most speciose and widely distributed, organized into nine clades and tribes (Lujan et al., 2015).

The genus *Ancistrus* Kner, 1854 (Hypostominae, Ancistrini) occurs from Panama to Argentina, presenting 65 valid species, in addition to distinct lineages not formally identified in the scientific literature due to its taxonomic complexity (Ferraris, 2007; Armbruster, 2008; Prizon et al., 2018; Borba et al., 2019; Fricke et al., 2021). From a chromosomal point of view, *Ancistrus* represents one of the most diverse lineages of Loricariidae, emphasizing their extensive variation in the diploid number (2n = 34 to 54, Supplementary Table S1). The vast majority of *Ancistrus* species have karyotypes with 2n ≤ 52, probably due to the result of Robertsonian (Rb) fusions (see Glugoski et al., 2020), whose occurrence has been evidenced through the *in situ* localization of repetitive sequences (Barros et al., 2017). The presence of sex chromosomes is another striking feature of chromosomal diversity in *Ancistrus*, with species showing simple systems (XX/XY, XX/X0 and ZZ/ZW; Mariotto et al., 2004; Alves et al., 2006; Mariotto and Miyazawa, 2006; de Oliveira et al., 2007), multiple (XX/XY_1_Y_2_ and Z_1_Z_1_Z_2_Z_2_/Z_1_Z_2_W_1_W_2_; de Oliveira et al., 2008) or absence of differentiated sex chromosomes (Supplementary Table S1).

Sex chromosomes have emerged independently in different fish lineages, evolving through alternative mechanisms and showing various degrees of heteromorphism, even among closely related species (Charlesworth et al., 2005; Cioffi et al., 2010; Henning et al., 2011; Cioffi et al., 2012; Cioffi et al., 2013). Sex chromosomes in *Ancistrus* have been evidenced by size heteromorphism and accumulation of heterochromatic regions (Mariotto et al., 2004; Alves et al., 2006; Mariotto and Miyazawa, 2006; de Oliveira et al., 2007; de Oliveira et al., 2008; de Oliveira et al., 2009). Recently, *in situ* localization of repetitive sequences have provided insights into the differentiation of these chromosomes in several groups of fish, including *Ancistrus* (Cioffi and Bertollo, 2010; Schemberger et al., 2014; Cioffi et al., 2014; Favarato et al., 2017; Prizon et al., 2018; Schemberger et al., 2019).

Repetitive sequences represent the largest portion of eukaryotic genomes and may be organized in tandem repeats (e.g., microsatellites and multigene families, like ribosomal DNAs) or dispersed (e.g., transposons and retrotransposons). Ribosomal DNAs (rDNA) are represented by two gene families: 45S ribosomal RNA genes (18S, 5.8S and 28S genes), and 5S ribosomal RNA genes (Long and Dawid, 1980). The mapping of these sequences has shown intense variation in the location and number of chromosomal sites in Loricariidae. The participation of repetitive sequences, including rDNA and sequences microsatellites in chromosomal rearrangements has been evidenced, showing the importance of these markers in comparative analyzes (Pansonato-Alves et al., 2013; Barros et al., 2017; Primo et al., 2017; Bueno et al., 2018; Pety et al., 2018; Glugoski et al., 2020; Santos da Silva et al., 2021). Simple short tandem repeats of generally 1-6 nucleotides, known as,microsatellites, constitute another important tandem repeats group (Martins, 2007). The microsatellites are abundant in eukaryotic genomes, commonly a heterochromatin component, but they could also be found in euchromatic regions (Martins, 2007; Cioffi et al., 2010; Santos da Silva et al., 2021). They are helpful cytogenetic markers to demonstrate minor chromosomal variations into related species groups, including birds, amphibians, and fish (Cioffi et al., 2010; de Oliveira et al., 2017; Da Silva et al., 2021). Furthermore, the participation of these sequences in breakpoints regions for chromosomal rearrangements and evolution of sex chromosomes has been shown in many species, including *Ancistrus* (Farré et al., 2011; Cioffi et al., 2017; Favarato et al., 2017; Prizon et al., 2017).

In the present work, we studied the karyotypes of two not formally described *Ancistrus* species (*Ancistrus* sp. 1 and *Ancistrus* sp. 2) from the Amazon region, using different groups of repetitive sequences, in order to understand their mechanisms of chromosomal diversification.

## Materials and Methods

### Samples

**FIGURE 1:**
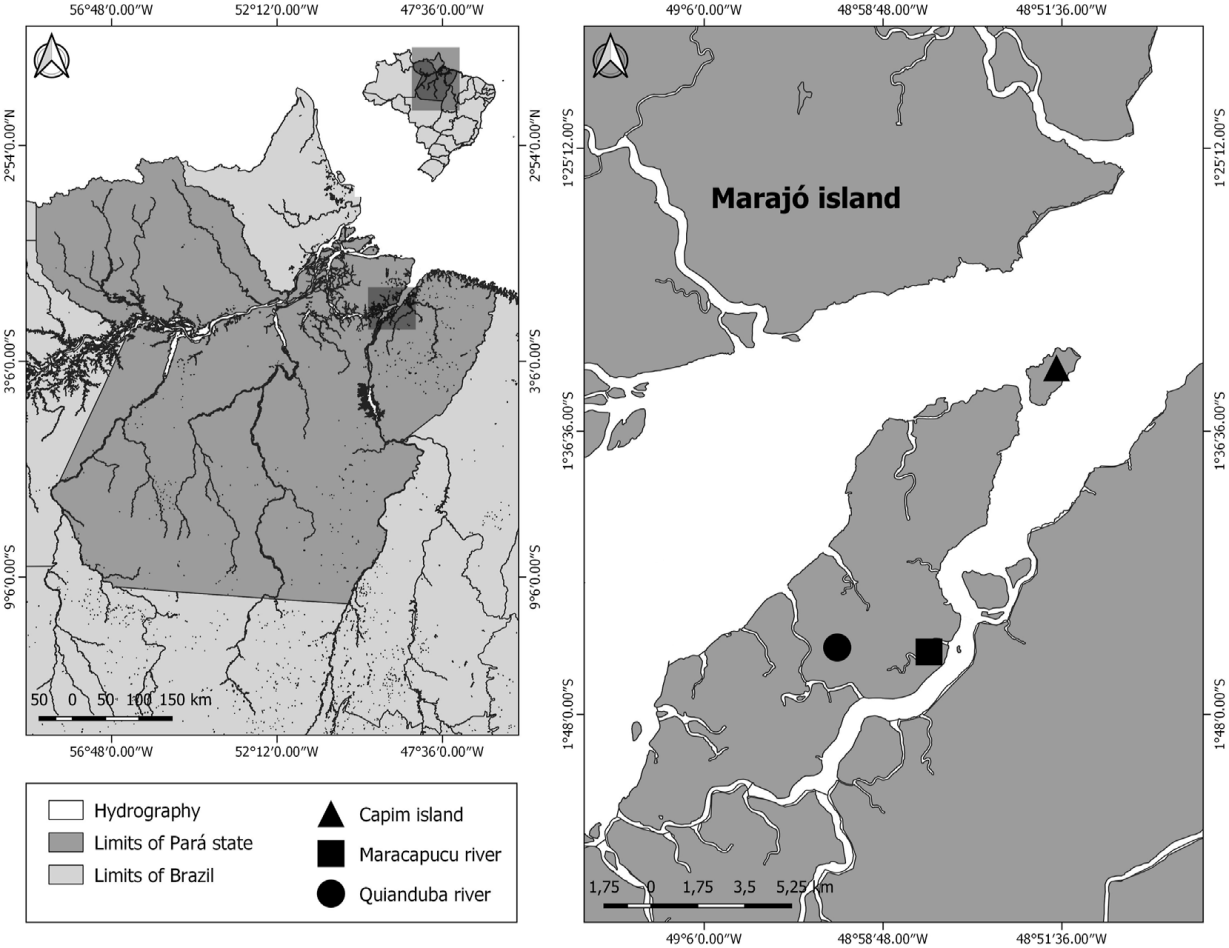
The geographical location of the collection points of specimens of Ancistrus species analyzed in this study. The map was built using Q-GIS version 3.4.5. The database was obtained from Instituto Brasileiro de Geografia e Estatística (IBGE).

Samples of two species of *Ancistrus* (*Ancistrus* sp. 1 and *Ancistrus* sp. 2) were analysed in this study. These species are morphologically different but are not yet described in the scientific literature. The samples were collected in distinct locations of the Tocantins-Araguaia River basin, in the Brazilian Amazon (Figure 1). Details about the collection points, number of individuals, and sex are presented in Table 1. The collection permit (number 13248) was issued by the Chico Mendes Institute for Biodiversity Conservation, Brazil. The Cytogenetics Laboratory of the Federal University of Pará had licenses for transport (number 19/2003) and the use of animals for this research (52/2003) as granted by the Ministry of the Environment. This study was approved by the Animal Ethics Committee of the Federal University of Pará (permission 68/2015). The specimens analyzed in this study were deposited in the Ichthyology Collection of the Center for Advanced Studies in Biodiversity (CEABIO/UFPA), Belém, Pará, Brazil.

**TABLE 1:** Sampling and collection sites of *Ancistrus* specimens analyzed in this study.

Species	Sex		River	Locality	Voucher	Coordinates	
*Ancistrus* sp. 1	5♂	2♀	A	Abaetetuba/PA	P4029	S01°45′18,2″	W49°00′38,8″
*Ancistrus* sp. 2	8♂	1♀	B	Abaetetuba/PA	P4263	S01°45′29,2″	W48°56′57″
	−♂	1♀	C	Abaetetuba/PA	P4251	S01°34′02,8″	W48°51′49,1″
Rivers: A—Quianduba River; B—Maracapucú River; C—Ilha do Capim; (−)—no samples							

### Chromosomal Analysis

Mitotic chromosomes were obtained from anterior kidney cells after *in vivo* treatment with colchicine solution (0.025%), as described by Bertollo et al. (1978). The animals were anesthetized with a eugenol solution (185 mg/L) (Vidal et al., 2008) and then sacrificed. Chromosomes were analyzed by classical staining (conventional staining with 5% Giemsa), C-banding (Sumner, 1972), and also molecular methods (Fluorescence *in situ* hybridization, FISH).

### Probes Labeling and Fluorescence *in situ* Hybridization

Genomic DNA was extracted using the PureLink Genomic DNA Kit (Promega), following the manufacturer’s instructions. We used two rDNA sequences for *in situ* localization experiments: an 18S rDNA probe (1,400 bp segment) isolated from *Ancistrus* sp. 1 genomic DNA according described in (Gross et al., 2010), and a 5S rDNA (GenBank accession no. MT018470) probe obtained from *Ancistrus aguaboensis* (Glugoski et al., 2020). rDNA probes were labeled by nick-translation with biotin or digoxigenin. Telomeric probes were PCR labeled with digoxigeninin-11-dUTP (Roche Applid Science^®^) using primers F-5′(TTAGGG)5-3′ and R-5′(CCCTAA)5-3′ without using template DNA (Ijdo et al., 1991). All PCR products were checked on 1% agarose gel electrophoresis. The nine microsatellite probes ((CA)n, (CAC)n, (CAG)n, (CG)n, (GA)n, (CAT)n, (GAA)n, (GAC)n and (TAA)n) were purchased already with direct labeling by Cy3 during synthesis. FISH was performed following the protocol proposed by Martins and Galetti (1999), with modifications, under the following stringency conditions: 2.5 ng/μL of each probe, 50% formamide, 2 x SSC, 10% dextran sulfate, and hybridization at 42°C for 16 h. Fluorescence signals were detected using Streptavidin Alexa Fluor 488 (Molecular Probes, Carlsbad, CA, United States) and anti-digoxigenin rhodamine Fab fragments (Roche Applied Science, Penzberg, Germany). Chromosomes were counterstained with 0.2 μg/ml 4′6-diamidino-2-phenylindole (DAPI) in Vectashield mounting medium (Vector, Burlingame, CA, United States).

### Image Capture and Analysis

Thirty metaphases per individual were analyzed to determine the diploid number, karyotype formula, and FISH experiments. Images of metaphases after Giemsa staining were obtained using an Olympus BX41 microscope (bright field) coupled to a CCD 1300QDS digital camera and analyzed using GenASIs ASI (Applied Spectral Imaging) software. FISH images were obtained using a Nikon H550S microscope and analyzed using Nis-Elements software. All images were adjusted using Adobe Photoshop CS6 software. The chromosome pairs were classified as metacentric (m), submetacentric (sm) and subtelocentric (st) following the criteria proposed by Levan et al. (1964). The count of the number of chromosome arms (Fundamental Number - FN) considered chromosomes m, sm and st as bi-armed.

## Results

### Classical Cytogenetics

**FIGURE 2:**
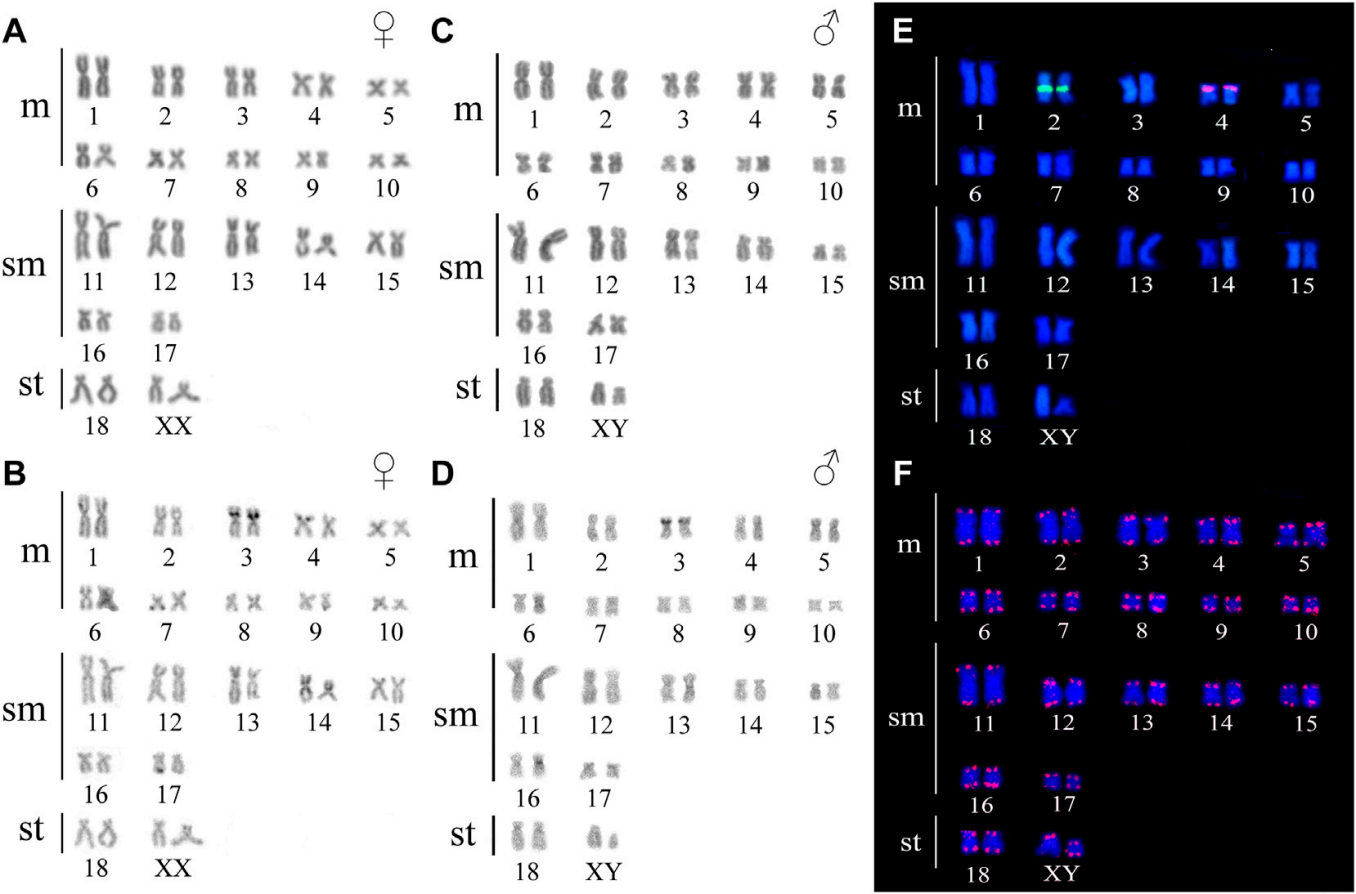
Karyotype of Ancistrus sp. 1. In **(A,B)** male karyotype stained with Giemsa and C-banding, respectively; in **(C,D)** female karyotype stained with Giemsa and C-banding, respectively; in **(E)** *in situ* localization of 18S rDNA (green) and 5S rDNA (red) sequences; in **(F)** *in situ* localization of telomeric sequences. The probes were labeled with FITC (green) and Cy3 (red), the chromosomes were counterstained with DAPI (blue).

*Ancistrus* sp. 1 demonstrated diploid chromosome number (2n), fundamental number (FN) and karyotype formula (KF) as follow: 2n = 38, FN = 72, KF = 20m + 14sm+2st. A heteromorphic chromosome pair was identified in males while the female karyotypes were homomorphic, characterizing a XX/XY sex chromosome system. The small subtelocentric Y chromosome and a medium X subtelocentric chromosome were recorded (Figures 2A,C). Constitutive heterochromatin (CH) occurred in a few regions in the karyotype, and was not evidenced in the sex chromosomes (Figures 2B,D).

**FIGURE 3:**
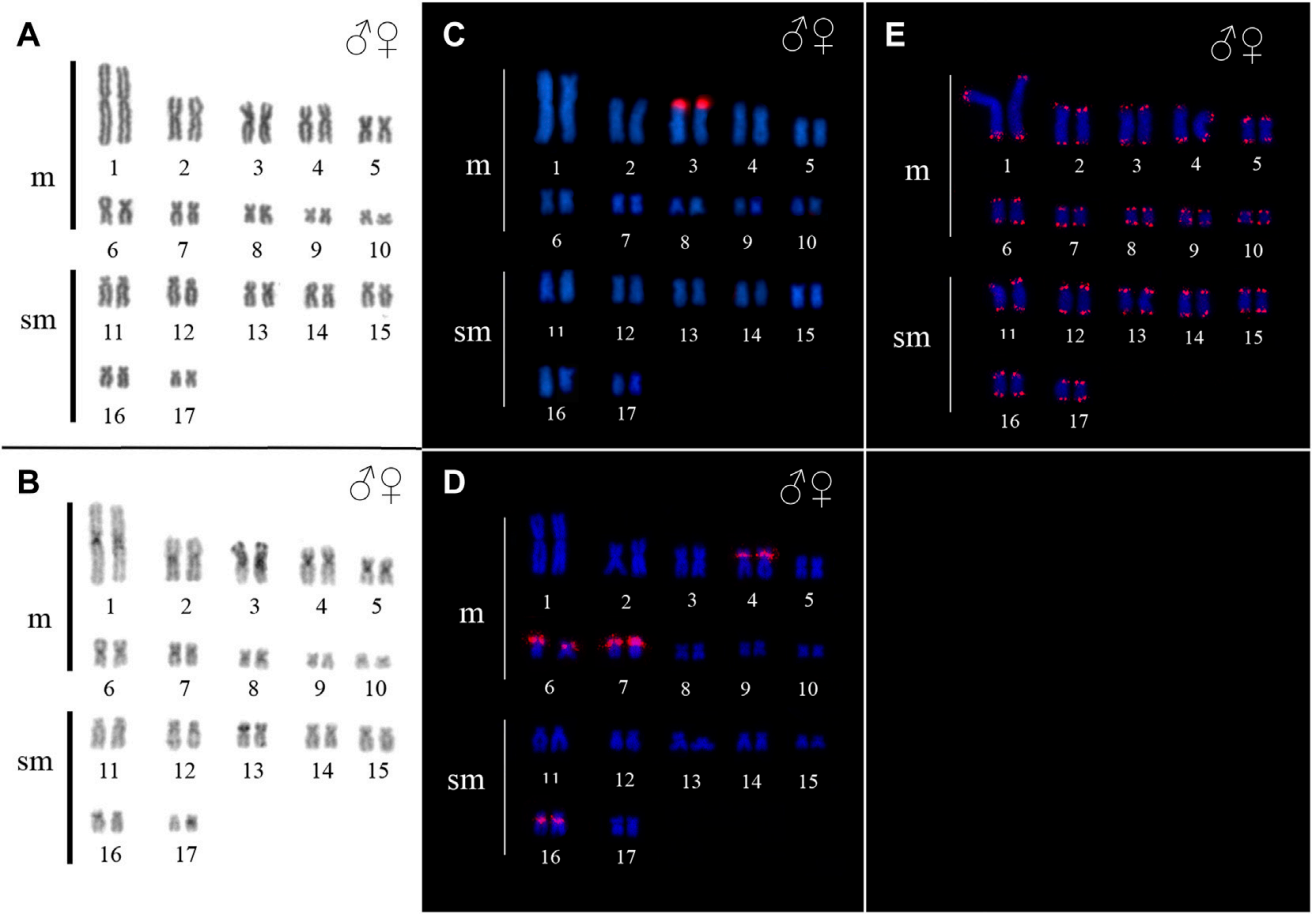
Karyotype of Ancistrus sp. 2. In **(A,B)** karyotype stained with Giemsa and C-banding, respectively; in **(C–E)** *in situ* location of 18S rDNA, 5S rDNA and telomeric sequences, respectively. The probes were labeled with Cy3 (red), the chromosomes were counterstained with DAPI (blue).

*Ancistrus* sp. 2 demonstrated diploid chromosome number (2n), fundamental number (FN) and karyotype formula (KF) as follow: 2n = 34, FN = 68, KF = 20m + 14sm. Additionally no morphologically differentiated of sex chromosomes was found (Figure 3A). Some heterochromatic blocks are mainly distributed in the centromeric and pericentromeric region of chromosomes 1, 2, 3, 4, 5, 6, 7, 8, 13, and 16, besides conspicuous blocks in the short arm (p) distal region of the chromosome 3, coincident with the 18S rDNA sites (Figures 3B,C).

### Molecular Cytogenetics

In *Ancistrus* sp. 1, the 18S rDNA site was located in the proximal region of the long arm (q) of pair 2, while the 5S rDNA occurred in the interstitial region of 4p (Figure 2E). In *Ancistrus* sp. 2, the 18S rDNA was located in the distal region of 3p (Figure 3C). The 5S rDNA demonstrated multiple sites located in the proximal region of the 4p and 6p, and pericentromeric in the 7p and 16q (Figure 3D). Interstitial telomeric sites (ITS) were not observed in any analyzed karyotypes (Figure 2F; Figure 3E).

**FIGURE 4:**
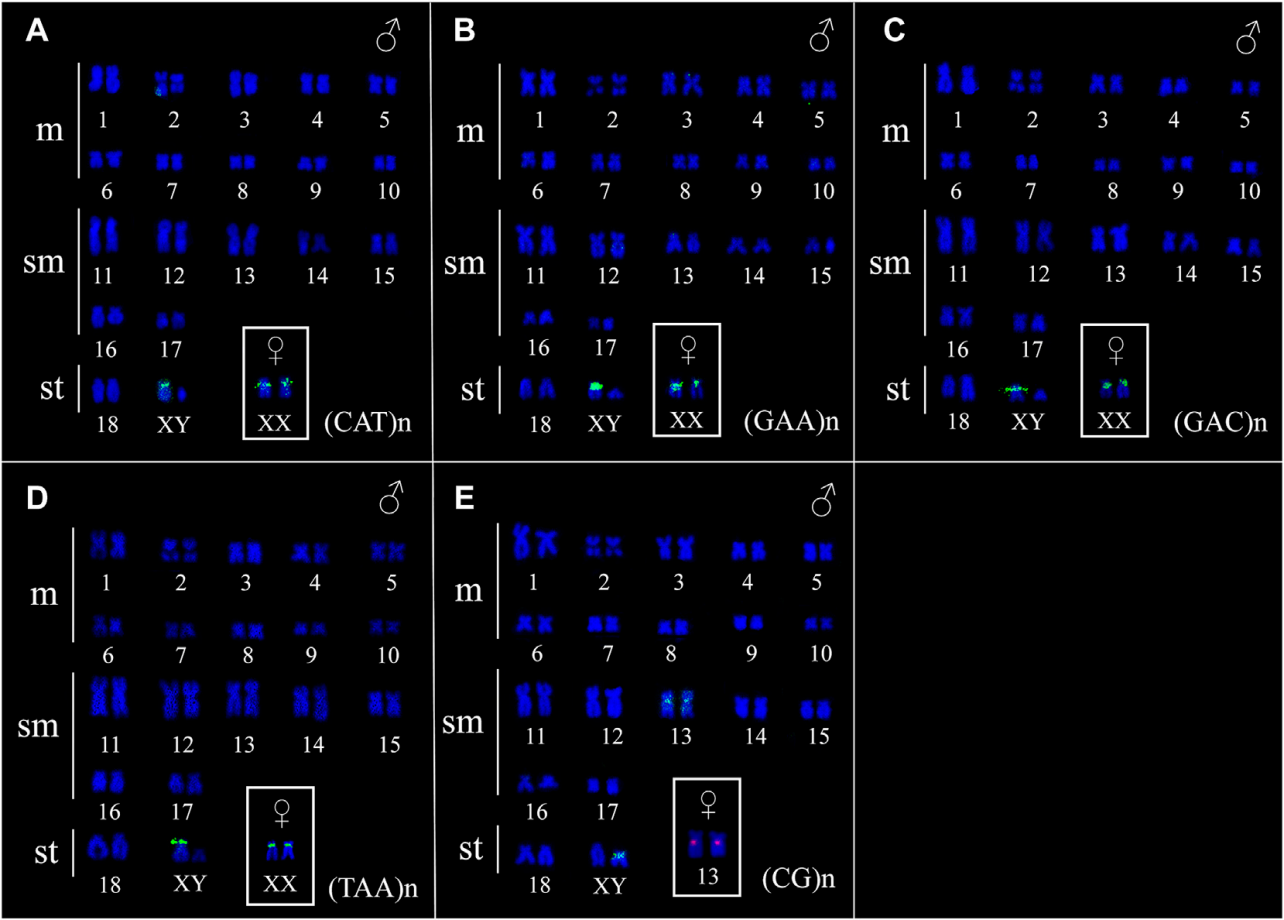
Fluorescence *in situ* hybridization indicating the physical location of microsatellite sequences in the karyotype of Ancistrus sp. 1: **(A)** (CAT)n, **(B)** (GAA)n, **(C)** (GAC)n, **(D)** (TAA)n, **(E)** (CG)n. Karyotypes of males are presented; female sex chromosomes are in the boxes. The probes were labeled with FITC (green) and with Cy3 (red), chromosomes were counterstained with DAPI (blue).

**FIGURE 5:**
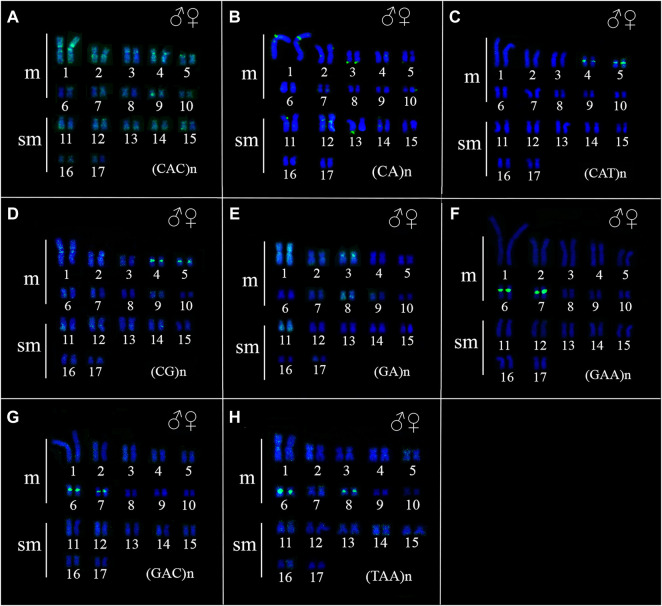
Fluorescence *in situ* hybridization indicating the physical location of microsatellite sequences in the karyotype of Ancistrus sp. 2: **(A)** (CAC)n, **(B)** (CA)n, **(C)** (CAT)n, **(D)** (CG)n, **(E)** (GA)n, **(F)** (GAA)n, **(G)** (GAC)n, **(H)** (TAA)n. The probes were labeled with FITC (green), the chromosomes were counterstained with DAPI (blue).

**FIGURE 6:**
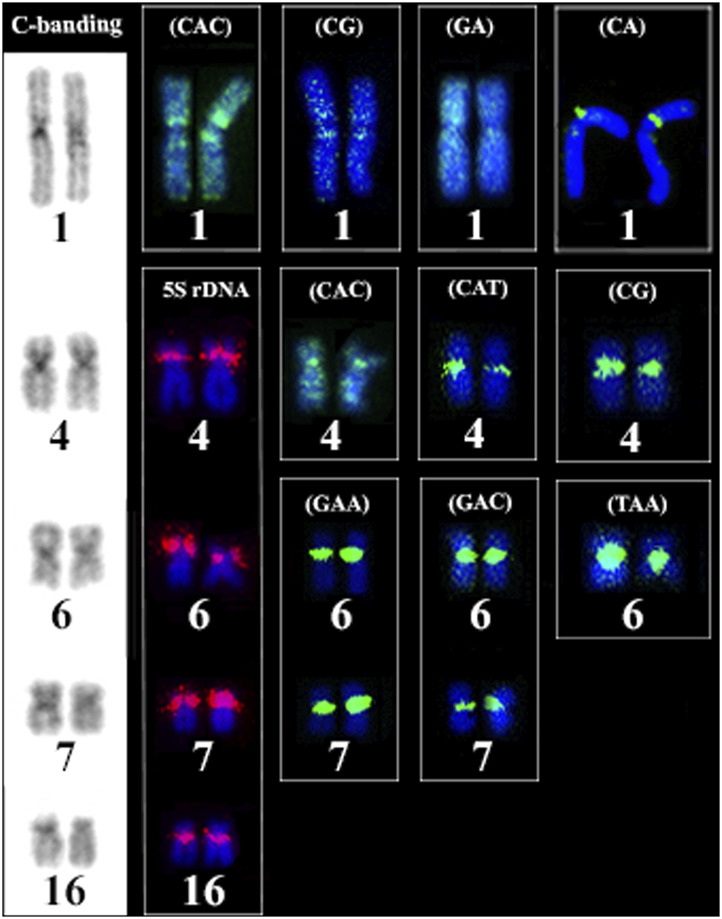
Comparative analysis of the distribution of heterochromatic regions, 5S rDNA sites and microsatellite sequences and in the karyotype of Ancistrus sp. 2. Boxes indicate the repetitive sequences in heterochromatic regions of chromosome pairs 1, 4, 6, 7 and 16 of *Ancistrus* sp. 2. Chromosomes stained with Giemsa; hybridization signals with FITC (green) and Cy3 (red), chromosomes were counterstained with DAPI (blue).

The FISH results of microsatellite sequences in the karyotypes of the two *Ancistrus* species were summarized in Table 2. In *Ancistrus* sp. 1, the microsatellites (CAT)n, (GAA)n, (GAC)n and (TAA)n were located exclusively in the pericentromeric region of the X chromosome; (CG)n showed signs in the pericentromeric region of the Y chromosome and in the centromeric region of the pair 13 in individuals of both sexes (Figures 4A–E). Microsatellites (CA)n, (GA)n, (CAC)n and (CAG)n did not demonstrate any hybridization signal in the karyotype of these species (Table 2). In *Ancistrus* sp. 2, the same sets of microsatellites were observed in other pairs of chromosomes, mainly in centromeric and pericentromeric regions associated with heterochromatic regions (Figures 5A–H, Figure 6). Some microsatellites, including (CAC)n, (CAT)n, (CG)n, (GAA)n, (GAC)n, and (TAA)n was associated with heterochromatic regions and coincident or adjacent to 5S rDNA sites in the pairs 4, 6 and 7 (Figure 6). The microsatellite (CAG)n did not show any hybridization signal in both karyotypes (Table 2).

**TABLE 2:** Comparative analysis of the presence/absence and location of microsatellite sequences between the karyotypes of the *Ancistrus* species analyzed in this study.

Microsatellites	Ancistrus sp. 1						Ancistrus sp. 2		
	Autosomes			Sex chromosomes		Autosomes			
	♂		♀	Chr. X	Chr. Y	♂		♀	
(CA)	−	−		−	−	+			+
(CG)	+	+		−	+	+			+
(GA)	−	−		−	−	+			+
(CAC)	−	−		−	−	+			+
(CAG)	−	−		−	−	−			−
(CAT)	−	−		+	−	+			+
(GAA)	−	−		+	−	+			+
(GAC)	−	−		+	−	+			+
(TAA)	−	−		+	−	+			+

(+)—presence of hybridization signal; (−)—no hybridization signal.

## Discussion

### Chromosomal Diversity in the Genus *Ancistrus*

The putative ancestral karyotype for Loricariidae presents 2n = 54, single nucleolus organizer regions (NOR) and few heterochromatic regions (Artoni and Bertollo, 2001; Ziemniczak et al., 2012). Nevertheless, considering the presence of 2n = 52 chromosomes in Pterygoplichthyini, the sister group for Ancistrini, Bueno et al. (2018) suggested that the putatively ancestral condition for Ancistrini is a diploid number of 52 chromosomes. Previously studies in *Ancistrus* have shown extensive chromosomal diversity with high variation in 2n values, the occurrence of multiple sites, as well as, 18S/5S rDNA synteny rupture (Supplementary Table S1). In this genus, species with lower 2n karyotypes have many chromosomes m/sm compared to those with higher 2n, which have more st/a chromosomes (Bueno et al., 2018; Glugoski et al., 2020). Thus, these findings suggests that chromosomal evolutions in this genus follows a tendency of 2n reduction due to the occurrence of Rb fusions (Barros et al., 2017).

Telomeric sequence mapping has shown the occurrence of ITS as a result of fusion events in Loricariidae (Barros et al., 2017; Primo et al., 2017). The species analyzed in this present study did not show the presence of ITS, which is in agreement with previous studies performed with other *Ancistrus* species (Prizon et al., 2018; Glugoski et al., 2020). The absence of ITS can be explained by the loss of these sequences during the fusion process (Slijepcevic, 1998).

Ribosomal genes have shown great diversity of location and number of sites among species and populations of different groups of fish (Gornung, 2013; Rebordinos et al., 2013). Mapping these sequences has revealed widely diverse chromosomal organizations in *Ancistrus* (Mariotto et al., 2011; Barros et al., 2017; Prizon et al., 2017; Prizon et al., 2018; Bueno et al., 2018; Glugoski et al., 2020). In Loricariidae, synteny between 18S/5S rDNA is considered a plesiomorphic character, with synteny break representing a derived state commonly observed in this group of fish (Bueno et al., 2018). This diversity of location and number of rDNA sites suggests the recurrent participation of these sequences in chromosomal reorganization events in Loricariidae (Pansonato-Alves et al., 2013; Prizon et al., 2016; Prizon et al., 2017; Barros et al., 2017; Prizon et al., 2018; Bueno et al., 2018; Santos da Silva et al., 2021).

Single 45S rDNA site represents a primitive character in Loricariidae (Artoni and Bertollo, 2001; Bueno et al., 2018). In the species described here and most of the analyzed *Ancistrus* species, these sequences are located in only one pair of chromosomes (see Supplementary Table S1), suggesting the maintenance of the primitive condition. Variations in the chromosomal position of these sequences were observed among the species in the present study, probably due to pericentric inversions.

Our results showed that the 5S rDNA was more dynamic than the 18S rDNA, varying in the number of sites among the analyzed karyotypes. This observation was consistent with previous observations performed in other *Ancistrus* species (Barros et al., 2017; Prizon et al., 2017; Glugoski et al., 2020; Supplementary Table S1). Studies have been showing that these sequences are involved in double-stranded DNA breaks and chromosomal rearrangements in *Ancistrus* (Mariotto et al., 2011; Favarato et al., 2016; Barros et al., 2017; Glugoski et al., 2020). Barros et al. (2017) demonstrated that the occurrence of multiple 5S rDNA sites is related to the emergence of pseudogenes in *Ancistrus* sp. (2n = 50). In addition, the involvement of 5S rRNA pseudogenes in Rb fusion events has been proposed in distinct genera of Loricariidae (Barros et al., 2017; Glugoski et al., 2018; Deon et al., 2020). Our results in *Ancistrus* sp. 2 showed multiple 5S rDNA sites located in the pericentromeric and proximal regions of some chromosome pairs. This data agrees with the hypothesis that 5S rDNA, or sequences derived from this gene family, may be involved in fusion events in *Ancistrus* sp. 2, as proposed previously for other species of this genus. Furthermore, these results supported the hypothesis that these sequences may represent evolutionary breakpoints regions (EBRs), which can be reused in chromosomal rearrangements in *Ancistrus* (Barros et al., 2017).

Comparative mapping of microsatellites revealed divergent patterns of organization between karyotypes of the *Ancistrus* species, occurring in euchromatic and heterochromatic regions in autosome and sex chromosomes. In *Ancistrus* sp. 2, the microsatellites mainly colonized heterochromatic blocks in centromeric and pericentromeric regions. Centromeric regions are characterized by the abundance of in tandem repeats, which are essential for maintaining the stability of this chromosomal region (Shang et al., 2010). However, the presence of different types of in tandem repeats, including microsatellites, at breakpoints for chromosomal rearrangements has been demonstrated previously (Kejnovsky et al., 2009; Cioffi and Bertollo, 2010; Ferré et al., 2011). The location of different microsatellites in the centromeric and pericentromeric region of metacentric chromosomes may indicate their association with EBRs (Ferré et al., 2011), suggesting the occurrence of Rb fusions during the evolution of the *Ancistrus* sp. 2. Furthermore, the association between microsatellites and rDNA sites has been observed in several organisms (Santos da Silva et al., 2021), corroborating to the chromosomal instability proposal to rDNA sites in *Ancistrus*. Therefore, the analyses carried out in this study suggest the participation of repetitive sequences in different mechanisms of chromosomal diversification in this group of neotropical fish.

### Sex Chromosomes in *Ancistrus*

In general, sex chromosomes occur in only a small portion of neotropical fish species, having independent evolutionary origins and evolving from different mechanisms (Charlesworth et al., 2005; Henning et al., 2008; Henning et al., 2011; Schamberger et al., 2019). In *Ancistrus*, extensive sex chromosome diversity is described, with different levels of morphological differentiation and DNA content (Mariotto et al., 2004; Mariotto and Miyazawa, 2006; de Oliveira et al., 2007; de Oliveira et al., 2008; de Oliveira et al., 2009). *Ancistrus* sex chromosomes have been analyzed mainly according to their size, heteromorphisms, and distribution of heterochromatic regions (Mariotto et al., 2004; Mariotto and Miyazawa, 2006; de Oliveira et al., 2007; de Oliveira et al., 2008; de Oliveira et al., 2009). Theoretically, heterochromatinization has been considered an essential step in proto sex chromosome differentiation due to the differential accumulation of repetitive sequences and its effects in decreasing the recombination rate (Cioffi et al., 2012). Partially or fully heterochromatic sex chromosomes could be considered a characteristic of well-differentiated systems in fish, as noted in *Eigenmannia*, *Tripohorteus*, *Characidium*, and Parodontidae (Henning et al., 2011; Cioffi et al., 2014; Schemberger et al., 2014; Ziemniczak et al., 2014; Pucci et al., 2016). However, the absence of heterochromatic regions is a frequent state in the sex chromosomes in *Ancistrus*, including XX/XY or ZZ/ZW systems (de Oliveira et al., 2007; de Oliveira et al., 2009; present study), suggesting that the sex chromosomes in *Ancistrus* evolved independently and, therefore, are at different stages of differentiation regarding the accumulation of repetitive sequences and heterochromatinization.

*In situ* localization of repetitive sequences represent an important approach for studying sex chromosome diversity and evolution in fish (Cioffi et al., 2010; Schemberger et al., 2014; Schemberger et al., 2019). In *Ancistrus*, this approach has been applied in some species (Favarato et al., 2017; Prizon et al., 2018), indicating the participation of different repetitive sequences in the sex chromosome differentiation (Prizon et al., 2018). Here, the microsatellite comparative *in situ* localization mapping highlights differences between heteromorphic X and Y sex chromosomes in *Ancistrus* sp. 1. Usually, in XX/XY sex chromosome systems, the heteromorphic sex chromosome differentiates by accumulating repetitive sequences and heterochromatinization (Charlesworth et al., 2005). Furthermore, the Y chromosome generally follows through a degeneration pathway due to the absence of recombination, leading to its reduced size (Charlesworth et al., 2005). In this study, a higher concentration of microsatellite sequences were found in the X when compared to the Y chromosome; however, the presence of Y-speceific microsatellite sequences was also observed. These results can be explained, in part, by partial recombination restriction between the homologs of the proto sex pair promoted by the differential accumulation of repetitive sequences (Kejnovsky et al., 2009).

Sex chromosomes are commonly rich in distinct families of transposable elements (TE) and in tandem repeats (Charlesworth et al., 2005). The invasion of sex chromosomes by TE occurs at different stages of the differentiation of these chromosomes (Charlesworth et al., 2005; Schemberger et al., 2019). These sequences are inactivated or degenerated leading to heterochromatinization of parts of the sex chromosomes (Charlesworth et al., 2005). In *Ancistrus* sp. 1, C-banding demonstrated the absence of heterochromatin in the sex chromosomes, suggesting a recent stage of differentiation when compared to sex chromosomes from other fish groups (Cioffi et al., 2010; Henning et al., 2011; Cioffi et al., 2014; Schemberger et al., 2014; Schemberger et al., 2019). On the other hand, heteromorphic size suggests the occurrence of Y chromosome degeneration in *Ancistrus* sp. 1, as demonstrated by the different repetitive DNA content between X and Y-chromosomes. Future analyses integrating *in situ* location data from different repetitive units classes and epigenetic analyses will be important to test the condition of repetitive DNA segments that have not undergone heterochromatinization in *Ancistrus* sex chromosomes.

## Conclusion

Our study provided additional evidence on the evolutionary pathways to 2n reduction in *Ancistrus* species, highlighting specific chromosomal features that have emerged throughout their life. The obtained data also suggest the participation of repetitive sequences acting in *Ancistrus* sp. 1 and *Ancistrus* sp. 2 diversification, as those sequences can be involved in the Robertsonian rearrangements and sex chromosomes differentiation.

## Data Availability

The original contributions presented in the study are included in the article/Supplementary Material, further inquiries can be directed to the corresponding author.
