# Supplementary material for: Chromosomal Diversification in Ancistrus Species (Siluriformes: Loricariidae) Inferred From Repetitive Sequence Analysis
Source: Front Genet. 2022 Mar 24;13:838462. doi: 10.3389/fgene.2022.838462 (PMC8987504; doi:10.3389/fgene.2022.838462)
Supplement: Supplementary file 1 [file DataSheet1.PDF]

Supplementary Material

1 Supplementary Table S1. Chromosomal diversity available in the literature and obtained in the present study for species of the genus *Ancistrus*. Data are arranged in ascending order of diploid number. Adapted from Glugoski et al. (2020).

Species	2n	FN	KF	SC	5S rDNA	18S rDNA	Ref.
Ancistrus sp. 2	34	68	20m+14sm	Not found	Multiple	Simple	U
Ancistrus cuiabae	34	68	20m+8sm+6st	Not found	Multiple	Simple	J
Ancistrus cuiabae	34	68	20m+8sm+6st	Not found	Unknown	Simple	F
Ancistrus cuiabae	34	67	19m+8sm+6st+1a	Not found	Unknown	Simple	F
Ancistrus cuiabae	34	66	18m+8sm+6st+2a	Not found	Unknown	Unknown	F
Ancistrus sp. “Purus”	34	68	♂21m+11sm+2st	XX/XY	Multiple	Simple	I, O
			♀20m+2sm+2st				
Ancistrus sp. “Catalão”	34	68	22m+8sm+4st	XX/XY	Multiple	Simple	O
Ancistrus sp. 1	38	72	20m+14sm+2st	XX/XY	Simple	Simple	U
Ancistrus n.sp. 1	38	76	30m/sm+8st	Not found	Unknown	Unknown	A
Ancistrus sp. “Trombetas”	38	73	22m+8sm+5st+3a	Not found	Unknown	Unknown	I
Ancistrus dubius	♀38	76	26m+10sm+2st	XX/XY1Y2	Simple	Simple	H, O
Ancistrus sp. “Balbina”	♂39	78	27m+10sm+2st				
Ancistrus n.sp. 1	♂39	78	33m+6sm	XX/X0	Unknown	Unknown	D
	♀40	80	34m+6sm				
Ancistrus sp. 13	40	80	26m+10sm+4st	Not found	Multiple	Simple	J
Ancistrus sp. 13	40	80	30m+6sm+4st	Not found	Unknown	Unknown	L
Ancistrus sp.	42	84	18m+16sm+8st	Not found	Multiple	Simple	P
Ancistrus cf. *dubius*	42	84	24m+10sm+8st	Not found	Unknown	Unknown	C
Ancistrus cf. *dubius*	42	84	24m+10sm+8st	XX/XY	Multiple	Simple	J
Ancistrus cf. *dubius*	42	84	24m+10sm+8st	XX/XY	Multiple	Simple	J
Ancistrus cf. *dubius*	42	84	24m+10sm+8st	XX/XY	Multiple	Simple	J
Ancistrus sp. “Vermelho”	42	78	26m+6sm+4st+6a	Not found	Unknown	Unknown	I
Ancistrus cf. *dubius*	44	72	18m+10sm+16st/a	ZZ/ZW	Unknown	Unknown	B
Ancistrus sp. 08	44	80	18m+10sm+8st+8a	ZZ/ZW	Multiple	Simple	J
Ancistrus maximus	46	♂81	18m+11sm+6st+11a	XX/XY	Simple	Simple	E, O
Ancistrus sp. “Macoari”		♀82	18m+12sm+6st+10a				
Ancistrus ranunculus	48	82	♂20m+8sm+6st+14a	ZZ/ZW	Simple	Simple	G, O
			♀19m+9sm+6st+14a				
Ancistrus abilhoai	48	90	22m+14sm+6st+6a	Not found	Simple	Simple	N
Ancistrus aguaboensis	50	80	16m+10sm+4st+20a	Not found	Multiple	Simple	S
Ancistrus tombador	50	84	14m+12sm+8st+16a	Not found	Unknown	Unknown	L
Ancistrus taunayi	50	92	22m+10sm+10st+8a	ZZ/ZW	Simple	Simple	M
Ancistrus cirrhosus	50	86	10m+14sm+12st+14a	Not found	Multiple	Simple	Q
Ancistrus sp. “Mourão River”	50	92	12m+18sm+12st+8a	Not found	Multiple	Simple	Q
Ancistrus sp. “São Francisco Verdadeiro River”	50	94	14m+16sm+14st+6a	Not found	Multiple	Simple	Q
Ancistrus sp. “Ocoí	50	94	10m+18sm+16st+6a	Not found	Multiple	Simple	Q

River”							
Ancistrus sp. “São Francisco Falso River”	50	94	10m+18sm+16st+6a	Not found	Multiple	Simple	Q
Ancistrus sp. “19 Stream”	50	92	♂11m+18sm+13st+8a<br>♀12m+18sm+12st+8a	XX/XY	Multiple	Simple	Q
Ancistrus sp. “Keller River”	50	92	♂11m+18sm+13st+8a<br>♀12m+18sm+12st+8a	XX/XY	Multiple	Simple	Q
Ancistrus sp.	50	88	20m+12sm+6st+12a	Not found	Multiple	Simple	R
Ancistrus sp. 06	50	86	18m+10sm+8st+14a	Not found	Simple	Simple	J, L
Ancistrus sp.	52	76	12m+10sm+30st/a	Not found	Simple	Multiple	K
Ancistrus n.sp. 2	52	84	32m/sm+20st/a	Not found	Unknown	Unknown	A
Ancistrus n.sp. 2	52	90	10m+16sm+12st+14a	Not found	Unknown	Simple	D
Ancistrus multispinnis	52	80	28m/sm+24st/a	Not found	Unknown	Unknown	A
Ancistrus cf. *multispinis*	52	84	16m+10sm+6st+20a	Not found	Multiple	Simple	S
Ancistrus sp. “Dimona”	52	78	16m+8sm+2st+26a	Not found	Unknown	Unknown	I
Ancistrus aff. *dolichopterus*<br>*Ancistrus* sp. “Piagaçu”	52	♂78<br>♀79	16m+8sm+2st+26a<br>16m+9sm+2st+25a	ZZ/ZW	Multiple	Simple	G, O
Ancistrus dolichopterus<br>*Ancistrus* sp. “Barcelos”	52	♂80<br>♀79	12m+12sm+4st+24a<br>11m+12sm+4st+25a	Z1Z1Z2Z2/ Z1Z2W1W2	Multiple	Simple	H, O
Ancistrus sp. 04	52	82	16m+8sm+6st+22a	Not found	Multiple	Simple	J, L
Ancistrus sp. 04	52	82	16m+8sm+6st+22a	Not found	Unknown	Unknown	L
Ancistrus sp. 01	54	84	14m+8sm+8st+24a	Not found	Unknown	Unknown	L
Ancistrus sp. 03	54	84	14m+8sm+8st+24a	Not found	Unknown	Unknown	L
Ancistrus claro	54	84	14m+8sm+8st+24a	Not found	Multiple	Simple	J, L

Legends: 2n – Diploid number; **FN** – Fundamental number; **KF** – Karyotypic formula; **SC** – Sex chromosomes; **Ref.** – References. **References**: **A** – Alves et al. (2003); **B** – Mariotto et al. (2004); **C** – Mariotto et al. (2006); **D** – Alves et al. (2006); **E** – de Oliveira et al. (2006); **F** – Mariotto et al. (2009); **G** – de Oliveira et al. (2007); **H** – de Oliveira et al. (2008); **I** – de Oliveira et al. (2009); **J** – Mariotto et al. (2011); **K** – Reis et al. (2012); **L** – Mariotto et al. (2013); **M** – Konerat et al. (2015); **N** – Ribeiro et al. (2015); **O** – Favarato et al. (2016); **P** – Prizon et al. (2016); **Q** – Prizon et al. (2017); **R** – Barros et al. (2017); **S** – Glugoski et al. (2020); **U** – Present Study.
